# Modified Lefort partial colpocleisis

**DOI:** 10.1007/s00192-020-04545-5

**Published:** 2020-10-01

**Authors:** Hongtao Lv, Fengnian Rong

**Affiliations:** Department of Gynecology and Obstetrics, The First Affiliated Hospital of Shandong First Medical University, Jinan, 250014 Shandong China

**Keywords:** Pelvic organ prolapse, LeFort colpocleisis, Vaginal vault prolapse

## Abstract

**Introduction and hypothesis:**

We present a surgical video that describes the technical considerations for performing a modified LeFort partial colpocleisis.

**Methods:**

Hydro-dissection with diluted pituitrin was performed before the creation of anterior and posterior mid-line incisions through which lateral flaps were created bilaterally to expose the bladder and rectum fascia. Several purse-string sutures were placed to push the bladder and rectum back to their normal positions and reinforce the fascia under the vaginal wall. After removing the excess part of the vaginal wall, the lateral margins were re-approximated to create lateral channels that were wide enough to fit one finger. Perineoplasty was then performed to reduce the length of the genital hiatus.

**Results:**

The procedure was performed in a 76-year-old woman with stage III vaginal vault prolapse (POP-Q C + 2), stage IV anterior prolapse (POP-Q Ba+5), stage II posterior prolapse (POP-Q Bp-1), and mild occult stress urinary incontinence. The patient recovered well postoperatively, without recurrent prolapse and/or stress incontinence during 6 months of follow-up.

**Conclusions:**

Our modified technique used traditional suture methods to strengthen the bladder and rectum fascia, keeping most of the vaginal wall to create a solid longitudinal septum in the center of the vagina that supported the vaginal vault.

**Electronic supplementary material:**

The online version of this article (10.1007/s00192-020-04545-5) contains supplementary material. This video is also available to watch on http://link.springer.com/. Please search for this article by the article title or DOI number, and on the article page click on ‘Supplementary Material’.

## Introduction

Pelvic organ prolapse (POP) is very common. It is estimated that nearly 50% of women will develop some form of POP, and 10–20% of these women seek medical assistance [1]. Surgical treatment options for POP are classified as either reconstructive or obliterative techniques. For older women who do not seek to maintain coital function, the LeFort partial colpocleisis is a representative obliterative surgical technique used for frail, older women with advanced apical POP who are inappropriate candidates for vaginal reconstructive surgery [2]. The reported rate of POP recurrence after LeFort partial colpocleisis is 4.2% [3]. It has been speculated that the main risk factors for recurrence are an increased postoperative vaginal length and a wide vaginal hiatus [4]. Herein, we describe our modified LeFort partial colpocleisis technique for POP, which strengthens the pelvic floor on three levels [5].

## Methods

A 76-year-old woman presented with a symptomatic vaginal bulge for 1 year and mild occult stress urinary incontinence (SUI). She had undergone an abdominal hysterectomy for uterine myoma. Examination revealed stage III vaginal vault prolapse (POP-Q C + 2), stage IV anterior prolapse (POP-Q Ba+5), and stage II posterior prolapse (POP-Q Bp-1). The patient opted to undergo modified LeFort partial colpocleisis.

Procedure:

1. General anesthesia was induced via a laryngeal mask before hydro-dissection was performed with diluted pituitrin.
2. A mid-line incision was made anteriorly about 0.5 cm from the vaginal cutoff and 2–3 cm from the external orifice of the urethra. A combination of sharp and blunt dissection was then used to separate the anterior vaginal epithelium from the underlying connective tissue to expose the bladder fascia.
3. While paying careful attention to hemostasis, three to four purse-string sutures were placed to return the bladder to its normal position. U-shaped sutures were then used to strengthen the fibrous connective tissue under the vaginal epithelium.
4. The same procedures were performed for the posterior vaginal epithelium. The upper edge of the incision in the posterior vaginal wall was 0.5 cm below the cutoff of the vagina, while the lower edge of the incision was about 2 cm from the perineum.
5. The excessive vaginal wall was removed, and the lateral margins were re-approximated to create a strong longitudinal septum in the mid-line of the vagina, so that the lateral channels were wide enough to fit one finger.
6. Two Allis clamps were placed on the genital hiatus before a diamond-shaped flap was removed. The perineal body was reconstructed using a series of interrupted sutures. The skin was then sutured subcutaneously with 3-0 absorbable suture.
7. A drainage strip was placed in the septum, and the lateral channels were each filled with gauze to prevent hematoma formation.

## Results

The operation time was 53 min, and the estimated blood loss was 220 ml. There were no intra- or postoperative complications. The urinary catheter was removed 3 days postoperatively. The patient had recovered well by 1 month postoperatively. There was no prolapse recurrence or SUI during 6 months of follow-up.

## Discussion

The present case suggests that Modified LeFort partial colpocleisis is a good treatment option for advanced POP in select older women, especially those with vaginal vault prolapse.

Following are some key points of the operation.

1. Colporrhaphy (level II) and perineoplasty (level III) were added to colpocleisis, creating a solid longitudinal vaginal septum to increase the support to the vaginal vault (level I).
2. The distal edge of the incision was closed transversely to reduce the tension on the urethra and minimize the risk of postoperative SUI.
3. Gauze and drainage strips were used to prevent postoperative hematoma, which could cause prolapse recurrence.

The advantage of the modified LeFort partial colpocleisis is that the defects of the three levels were simultaneously repaired. The disadvantage of this technique is that a small number of patients may require additional treatment for postoperative SUI [6]. However, most de novo urinary incontinence is mild and does not require intervention [7]. A subsequent surgery is required in 13% of women with occult SUI undergoing POP-only surgery, and there are no differences in the global impression of improvement and quality of life between women who require a mid-urethral sling and those who do not [6]. In addition, our procedure reserves a 2–3 cm length of vaginal wall at the mid-urethral level for the potential addition of postoperative tension-free vaginal tape.

## Electronic supplementary material

ESM 1 (MP4 101,311 kb)
